# Sox9 Transcriptionally Represses *Spp1* to Prevent Matrix Mineralization in Maturing Heart Valves and Chondrocytes

**DOI:** 10.1371/journal.pone.0026769

**Published:** 2011-10-26

**Authors:** Jacqueline D. Peacock, Danielle J. Huk, Hasini N. Ediriweera, Joy Lincoln

**Affiliations:** 1 Molecular and Cellular Pharmacology Graduate Program, Leonard M. Miller School of Medicine, University of Miami, Miami, Florida, United States of America; 2 Center of Cardiovascular and Pulmonary Research, The Research Institute at Nationwide Children's Hospital, Department of Pediatrics, The Ohio State University, Columbus, Ohio, United States of America; 3 Medical Education, Leonard M. Miller School of Medicine, University of Miami, Miami, Florida, United States of America; 4 Department of Molecular and Cellular Pharmacology, Leonard M. Miller School of Medicine, University of Miami, Miami, Florida, United States of America; 5 Department of Medicine, Leonard M. Miller School of Medicine, University of Miami, Miami, Florida, United States of America; University of Bergen, Norway

## Abstract

Sox9 is an SRY-related transcription factor required for expression of cartilaginous genes in the developing skeletal system and heart valve structures. In contrast to positively regulating cartilaginous matrix, Sox9 also negatively regulates matrix mineralization associated with bone formation. While the transcriptional activation of Sox9 target genes during chondrogenesis has been characterized, the mechanisms by which Sox9 represses osteogenic processes are not so clear. Using ChIP-on-chip and luciferase assays we show that Sox9 binds and represses transactivation of the osteogenic glycoprotein *Spp1*. In addition, Sox9 knockdown in post natal mouse heart valve explants and rib chondrocyte cultures promotes *Spp1* expression and matrix mineralization, while attenuating expression of cartilage genes *Type II Collagen* and *Cartilage Link Protein*. Further, we show that Spp1 is required for matrix mineralization induced by Sox9 knockdown. These studies provide insights into the molecular mechanisms by which Sox9 prevents pathologic matrix mineralization in tissues that must remain cartilaginous.

## Introduction

The vertebrate skeletal system is primarily composed of cartilage and bone, formed during embryonic development. Most bones develop from replacement of cartilage through a process known as endochondral ossification [1], [2]. Cartilage formation is initiated with condensation of undifferentiated mesenchyme progenitor cells followed by proliferation and differentiation into immature chondrocytes. As development progresses, chondrocytes begin to secrete extracellular matrix (ECM) typical of cartilage including type II collagen (Col2a1), before passing through a transition stage to become hypertrophic [3]. In addition to increasing in volume, hypertrophic chondrocytes secrete ECM that is later mineralized. These cells then die, leaving behind a calcified matrix that is eventually broken down, allowing invasion of blood vessels and infiltration of bone-resorbing (osteoclasts) and bone-forming (osteoblasts) cells. Many key regulators have been shown to be required for cartilage and bone forming processes, including the transcription factor Sox9.

Sox9 is an SRY-related hydroxymethylglutaryl (HMG) box member of the Sox family and has been shown to regulate many developmental events [4]. In the developing cartilage, *Sox9* is expressed in immature chondrocytes and its function is required for mesenchyme cell condensation, proliferation and differentiation [1], [4]. This positive role of Sox9 during early stages of chondrogenesis is largely attributed to its ability to bind and transactivate SRY consensus sites within cartilage ECM genes; these include *Col2a1*, *Col9a2, Col11a2, Col27a1* and the proteoglycans *Aggrecan* and *Cartilage Link Protein* (*CLP*) [5], [6], [7], [8], [9], [10]. In vivo, mice with reduced *Sox9* function fail to develop cartilage [11], [12], [13] and mimic the human skeletal disorder, Campomelic Dysplasia [13], [14], [15], further supporting a positive role for Sox9 in promoting formation of cartilaginous connective tissue.

Following chondrocyte maturation, *Sox9* expression is downregulated and levels remain low during stages of terminal differentiation and matrix mineralization [4]. Enforced expression in mature chondrocytes results in delayed endochondral ossification [16], suggesting that Sox9 has a repressive role in bone formation. Consistently, *Sox9* haploinsufficiency leads to premature mineralization in many skeletal tissues [13]. Despite these observations, the mechanisms of Sox9 function in bone are not fully understood. In contrast to *Sox9*, expression of the transcription factor *Runx2* (*cfba1*) is high in mature chondrocytes and osteoblasts [4], [17], [18], [19]. It has previously been shown that Sox9 binds RUNX2 to prevent transactivation of RUNX2 target genes, including the glycoprotein, *Spp1* [20], thereby repressing bone formation. An additional mechanism of Sox9-mediated repression of osteogenic processes includes findings by Hattori et al., [16] showing that Sox9 negatively regulates *Vegfa* to inhibit cartilage vascularization and subsequent mineralization. Together these previous studies have revealed pivotal roles for Sox9 in regulating formation of cartilage and bone connective tissues in the skeletal system.

In addition to the skeletal system, we, and others have shown that Sox9 is also expressed in the developing heart [21], [22]. More specifically, Sox9 is highly expressed in a population of mesenchyme cells within the endocardial cushions that later give rise to mature valve structures [23], [24]. During early stages of endocardial cushion formation, Sox9 is required to expand the pool of proliferating mesenchyme precursor cells [21]. Following endocardial cushion formation, *Sox9* expression is maintained in valve primorida and studies in mice suggest that similar to functions in the skeletal system, Sox9 promotes cartilaginous matrix phenotypes [23]. Remodeling heart valves from embryonic *Col2a1cre;Sox9^fl/fl^* mice express significantly reduced levels of Col2a1 and CLP [21], while valves from viable *Col2a1cre;Sox9^fl/+^*adult mice are calcified and express high levels of osteogenic genes including *Runx2* and *Spp1* [21], [25]. This valvular phenotype in *Sox9* mutant mice is consistent with human calcific valve disease, frequently described as a pathological ‘bone-like’ process [26]. However, the molecular mechanism by which Sox9 prevents pathological matrix mineralization in normal cartilaginous heart valve connective tissue has not been examined.

In this study, we use a genome-wide microarray technique to screen for interactions with novel target gene DNA enriched by Sox9 chromatin immunoprecipitation (ChIP) in primary neonate limb tissue. ChIP-on-chip analysis revealed Sox9 binding to over 450 promoters, including a region approximately 5000 bp upstream of the osteogenic glycoprotein, *Spp1*. At the functional level, luciferase assays show that Sox9 significantly represses *Spp1* transactivation through interaction with an SRY binding site. Loss of *Sox9* function in primary heart valve explants and chondrocyte cultures increases *Spp1* transcript levels, while expression of chondrogenic matrix genes including *Col2a1* and *CLP* are decreased. Conversely, Sox9 gain of function promotes *Col2a1* and *CLP* expression. Finally, we show that *Spp1* function is required for matrix mineralization induced by Sox9 knockdown in these culture systems. Taken together, these data suggest that in maturing heart valves and chondrocytes, Sox9 negatively regulates matrix mineralization through repressive regulation of *Spp1*.

## Results

### Identification of Sox9 binding to Spp1 by ChIP-on-chip analysis

Our previous work has shown that several molecular phenotypes and signaling pathways are common to the skeletal system and heart valves. Similarly, Sox9 positively promotes cartilage and negatively regulates matrix mineralization in both these systems [13], [21], [23], [25]. While the mechanisms of Sox9 during chondrogenic processes have been well described, its molecular function in mineralized connective tissues is less well understood. To address this we performed ChIP-on-chip analysis using pooled Sox9 antibodies to enrich DNA/Sox9 complexes in chromatin isolated from post natal mouse limb tissue, a time point when endochondral ossification is active. Compared to IgG controls, over 450 promoter regions showed enrichment of Sox9 binding at levels comparable, or greater than the known target gene, *CLP* [8] (Table S1). From this array of findings we focused our study on the observed enrichment of Sox9 binding approximately 5000 bp upstream of the transcriptional start site of *Spp1* (Osteopontin), a known regulator of osteoblast differentiation and mineralized matrix deposition in vitro [27], [28].

**Figure 1:**
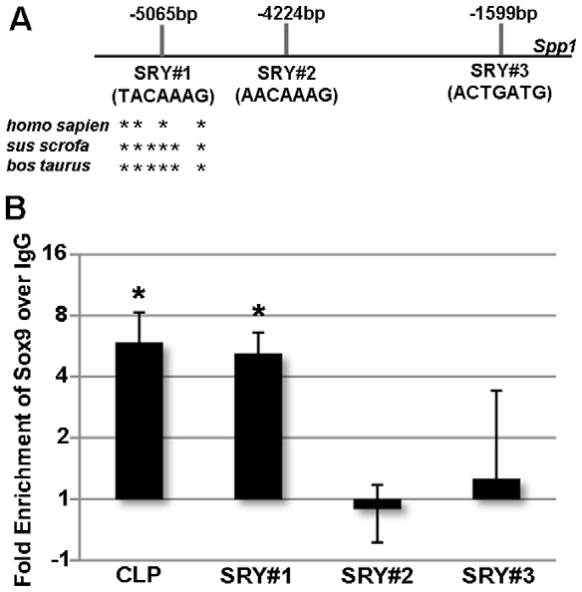
ChIP-on-chip analysis of Sox9 binding *Spp1*. (A) Schematic to show representation of canonical SRY binding sites as identified by Genomatix software within the 5’ region of *Spp1*. * indicate nucleotides conserved across species. (B) qPCR analysis to show fold enrichment of DNA/Sox9 complexes on SRY binding sites within the *Cartilage Link Protein* (*CLP*) and *Spp1* promoters. Fold changes in enrichment are shown for three independent samples following pulldown with pooled Sox9 antibodies, over IgG negative controls. *Student's t−test<0.05 compared to IgG.

Using Genomatix MatInspector software, we identified three canonical SRY binding sites within the 5000 bp candidate enhancer region of *Spp1*, denoted SRY#1 (−5065 bp), SRY#2 (−4224 bp) and SRY#3 (−1599 bp) (Figure 1A). In order to determine which SRY site(s) Sox9 binds to, and validate ChIP-on-chip observations, we performed three independent ChIP experiments and tested for enrichment following Sox9 IP within regions containing the putative SRY sites using specific primers. In these additional ChIP experiments, expected enrichment of Sox9 binding over IgG controls was observed at a known functional SRY binding site within the *CLP* promoter [8] (Figure 1B). In addition, enriched Sox9 binding was also observed within the *Spp1* enhancer region containing SRY#1 (−5065 bp) (Figure 1). In contrast, significant enrichment was not observed within regions containing SRY#2 (−4224 bp) or SRY#3 (−1599 bp). These studies suggest that Sox9 molecularly interacts with an SRY binding site within the 5’ enhancer of *Spp1.*

### Sox9 represses Spp1 promoter activity

**Figure 2:**
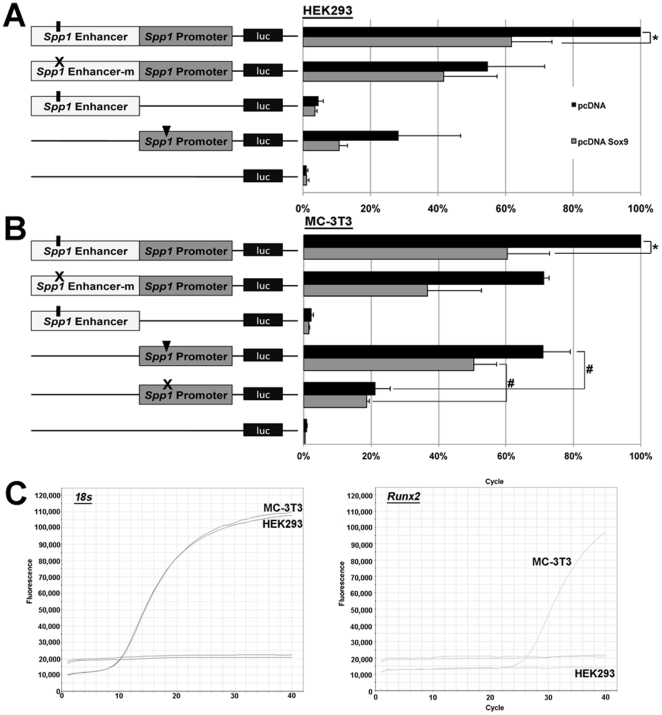
Luciferase assays of Sox9 function on *Spp1* activity. HEK293 (A) and MC-3T3 (B) cells were co-tranfected with 200 ng of the luciferase reporter construct pGL3 containing the *Spp1* promoter region (no SRY binding sites) (pGL3-*spp1*p), the 1599 bp 5’ *Spp1* enhancer region containing SRY#1 (pGL3-*spp1*En), or both the enhancer and promoter regions (pGL3-*spp1*), along with 200 ng of empty pcDNA (grey bars) or pcDNA containing full length murine Sox9 (pcDNA-Sox9, black bars). In addition, cells were co-transfected with 20 ng of pGL4 for normalization. Luciferase activity of pGL3-*spp1* with empty pcDNA was set to 100%. Note repression of pGL3-*spp1* activity (63%±8% (A) and 60.3%±12.6% (B) in the presence of pcDNA-Sox9. Cells were also co-transfected with pcDNA or pcDNA-Sox9 in the presence of pGL3-*spp1* in which the SRY#1 had been mutated from TACAAAG to TA*GCAG*G (pGL3-*spp1*mut). Note that pcDNA-Sox9 could not significantly repress pGL3-*spp1*mut unlike pGL3-*spp1*. *Student's t−test<0.05 compared to pcDNA. (C) qPCR-derived melting curves to show amplification of endogenous *18* *s* and *Runx2* expression levels in HEK293 and MC-3T3 cells.

To determine if the observed binding of Sox9 to the *Spp1* enhancer region is functionally important, luciferase assays were performed in HEK293 cells that express low levels of *Sox9* and *Spp1,* and MC-3T3 cells; a pre-osteoblastic cell line previously used to examine transcriptional regulation of *Spp1* [29]. In this experiment, HEK293 and MC-3T3 cells were co-transfected with either empty pcDNA or pcDNA-Sox9 in the presence of pGL3-*spp1*; consisting of the 1599 bp 5’ *Spp1* enhancer region containing SRY#1 in front of the published 826 bp *Spp1* promoter region [30]. The normalized luciferase activity in cells co-transfected with pGL3-*spp1* and empty pcDNA (pcDNA) was set at 100% and considered ‘baseline’ of pGL3-*spp1* activity in the two cell lines (Figure 2A, 2B, top black bar). In contrast to pcDNA, co-transfection with pcDNA-Sox9 consistently repressed pGL3-*spp1* activity by 63%±8% in HEK293 cells, and 60.3%±12.6% in MC-3T3 cells (Figure 2A, 2B, top grey bar). To examine the specificity of Sox9 mediating this repression through SRY#1, site directed mutagenesis was used to replace the Sox9/SRY core binding sequence (A/T A/T CAA A/T) site from TACAAAG to TA*GCAG*G (pGL3-*spp1*mut) [31]. The repressive effect of pcDNA-Sox9 on pGL3-*spp1* activity was not observed using the pGL3-*spp1*mut construct, suggesting that Sox9-mediated repression of *Spp1* is through SRY#1. Worthy of mention, pcDNA-Sox9 had no effect on activity of pGL3-empty or the *spp1* enhancer region (pGL3-*spp1*En) alone. However, pGL3-*spp1*p activity was significantly increased in MC-3T3 but not HEK293 cells, independent of SRY binding sites and exogenous Sox9 expression levels (pcDNA vs. pcDNA-Sox9). As this *Spp1* promoter region has previously been shown to be regulated by a functional Runx2 binding site, luciferase assays were repeated in MC-3T3 cells using a pGL3-*spp1*p mutant construct in which the Runx2 binding site had been mutated from ACCACA to AGGAGA (pGL3-*spp1p*mut) [30]. Activation of the *spp1* promoter was significantly diminished following mutation of Runx2 binding sites. This Runx2-mediated activation of the *spp1* promoter region is consistent with high levels of endogenous *Runx2* in MC-3T3, but not HEK293 cells (Figure 2C). In summary, findings from these luciferase assays show that Sox9 moderately represses *Spp1* promoter activity.

### Expression patterns of Sox9 and Osteopontin are mutually exclusive in post natal heart valves and limbs

**Figure 3:**
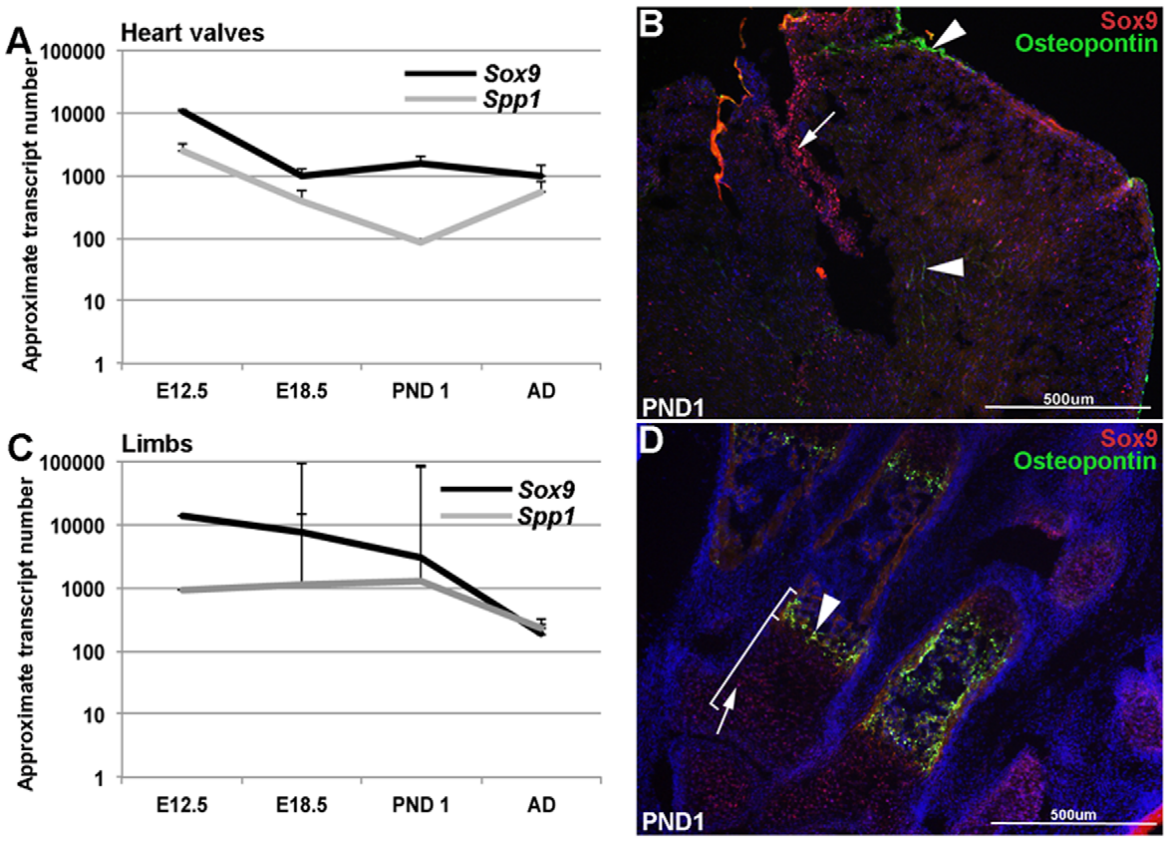
Temporal and spatial expression of Sox9 and Osteopontin in post natal heart valves and limbs. (A, C) qPCR analysis shows comparatively higher levels of *Sox9* compared to *Spp1* at all time points in post natal heart valves and limbs. For both genes, expression is higher during embryonic development compared to post natal and adult stages. (B, D) Immunohistochemistry reveals mutually exclusive expression patterns of Sox9 and Osteopontin in post natal tissues. In the heart (B), Sox9 is most highly detectable in heart valve structures (tricuspid valve shown) (arrow), while Osteopontin expression is observed in the subepicardial region and extracellular matrix within the myocardium (arrowheads). In the limb (D), the domain of Sox9 expression in developing chondrocytes (arrow) is distinct from that of Osteopontin in adjacent hypertrophic chondrocytes (arrowhead).

Previous studies by our group and others have shown that Sox9 and Spp1 play important roles in developing heart valves and limbs [12], [21], [25], [27]. Further, findings in this study suggest that Sox9 molecularly interacts with (Figure 1) and negatively regulates (Figure 2) *Spp1*. To support these observations, the temporal and spatial expression patterns of Sox9 and Spp1 were examined in heart valves and limbs using qPCR and immunohistochemistry (Figure 3). In both tissues, *Sox9* and *Spp1* are most highly expressed at E12.5 with a gradual decline to lower levels of expression by post natal and adult (3 month) stages (Figure 3A, 3C). As expected for a negative regulator, *Sox9* expression is consistently higher than *Spp1* at all time points (Figure 3A, 3C). Spatially, immunohistochemistry reveals that in both post natal hearts and limbs, Sox9 and Osteopontin (the protein product of *Spp1*) expression does not overlap, consistent with the notion that Sox9 functions to repress Osteopontin expression. In the heart, Sox9 is highly detectable within valvular structures (tricuspid valve shown), while Osteopontin expression is restricted to the subendocardial region and myocardial ECM (Figure 3B): a region in which Sox9 expression was not detected. In the limbs, Sox9 expression is consistent with developing chondrocytes [11] (Figure 3B), while Osteopontin expression is excluded from this expression domain [32]. These expression studies suggest that spatially, expression of Sox9 and Osteopontin are mutually exclusive in post natal heart valves and limbs.

### Sox9 loss of function in chondrocyte cultures and heart valve explants promotes Spp1 and represses cartilage gene expression

**Figure 4:**
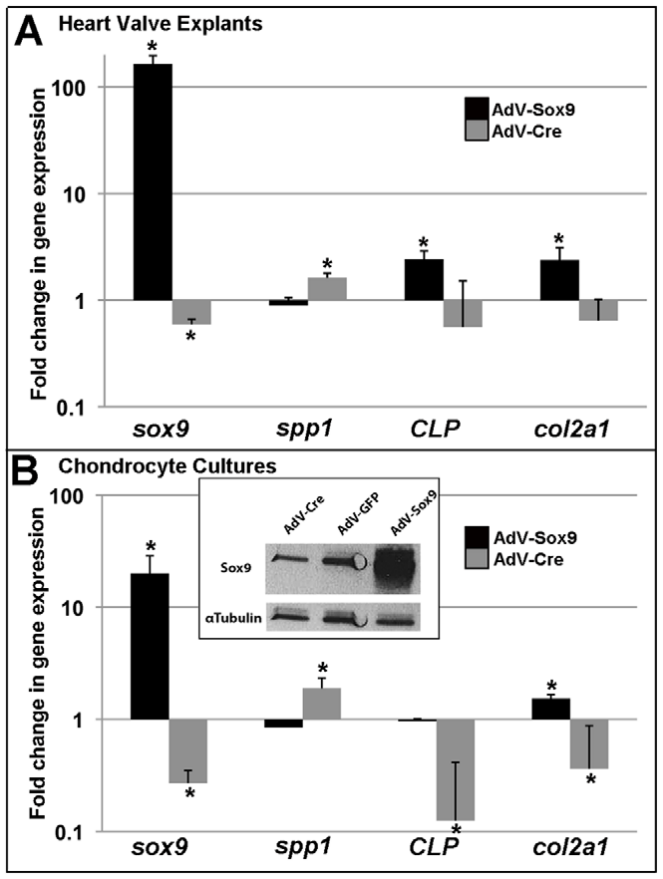
Effects on bone and cartilage gene expression in heart valve and chondrocyte cultures following Sox9 manipulation. Mitral valve explants (A) and chondrocyte cultures from post natal Sox9*^fl/fl^* mice (B) were infected for 4 hours with 2.5×10^10^ PFU/10 cm^2^ adenovirus targeting GFP (AdV-GFP), *Cre* (Adv-Cre) (grey bars), or full length murine Sox9 (AdV-Sox9) (black bars) to target knockdown or overexpression respectively. After 72 hours of initial infection, RNA was isolated and qPCR performed to examine changes in expression of *Sox9, Spp1* and cartilage genes *CLP* and *Col2a1* compared to AdV-GFP controls. Note logarithmic scale. *Student's t−test<0.05 compared to AdV-GFP. (B inset) Western blot analysis to show Sox9 expression levels in AdV-Cre, AdV-GFP and AdV-Sox9 treated chondrocytes. α-tubulin expression is included as a loading control.

To determine the effects of Sox9 function on *Spp1* expression, primary cultures were derived from post natal mouse rib chondrocytes or mitral valve explants and treated with adenovirus to target Sox9 function. Gene knockdown was achieved by infecting primary cultures isolated from *Sox9^fl/fl^* mice with adenovirus (AdV) producing Cre recombinase (AdV-Cre), while infection with AdV containing full length murine Sox9 (AdV-Sox9), served as an overexpression model [25]. As a control, cultures were infected with an equal titer of GFP-producing adenovirus (AdV-GFP) that did not alter Sox9 expression (data not shown). Compared to endogenous levels (AdV-GFP), *Sox9* expression was increased (164-fold±32.13, 20-fold±8.7) and decreased (0.59-fold±0.07, 0.27-fold±0.08) at transcript and protein levels following AdV-Sox9 and AdV-Cre treatments respectively in heart valve explants (Figure 4A) and chondrocyte cultures (Figure 4B). In addition to reducing *Sox9,* AdV-Cre significantly increased *Spp1* expression in both culture systems and reduced *CLP* and *Col2a1* expression in chondrocytes (Figure 4B). *CLP* and *Col2a1* expression levels were not altered in treated heart valve explants despite our previous studies showing downregulation in valves from embryonic *Col2a1cre;Sox9^fl/fl^* mice. This discrepancy is likely due to differences in endogenous *CLP* and *Col2a1* expression levels between the in vivo and in vitro systems. In contrast to AdV-Cre, AdV-Sox9 treatment in heart valve explants significantly increased expression of *CLP* and *Col2a1* (Figure 4A), and similarly in chondrocyte cultures, *Col2a1* was increased (Figure 4B). Although trends toward decreased *Spp1* expression were observed following AdV-Sox9 infection, significant changes were not achieved. This could be due to the low basal levels of *Spp1* in these culture systems prior to adenoviral infection (Figure 3A, 3C, data not shown). Collectively, these Sox9 targeting experiments show that Sox9 function plays pivotal roles in regulating cartilage and osteogenic gene expression in cultured heart valves and chondrocytes.

### Spp1 is required for Sox9-mediated calcification of heart valve and chondrocyte connective tissue

**Figure 5:**
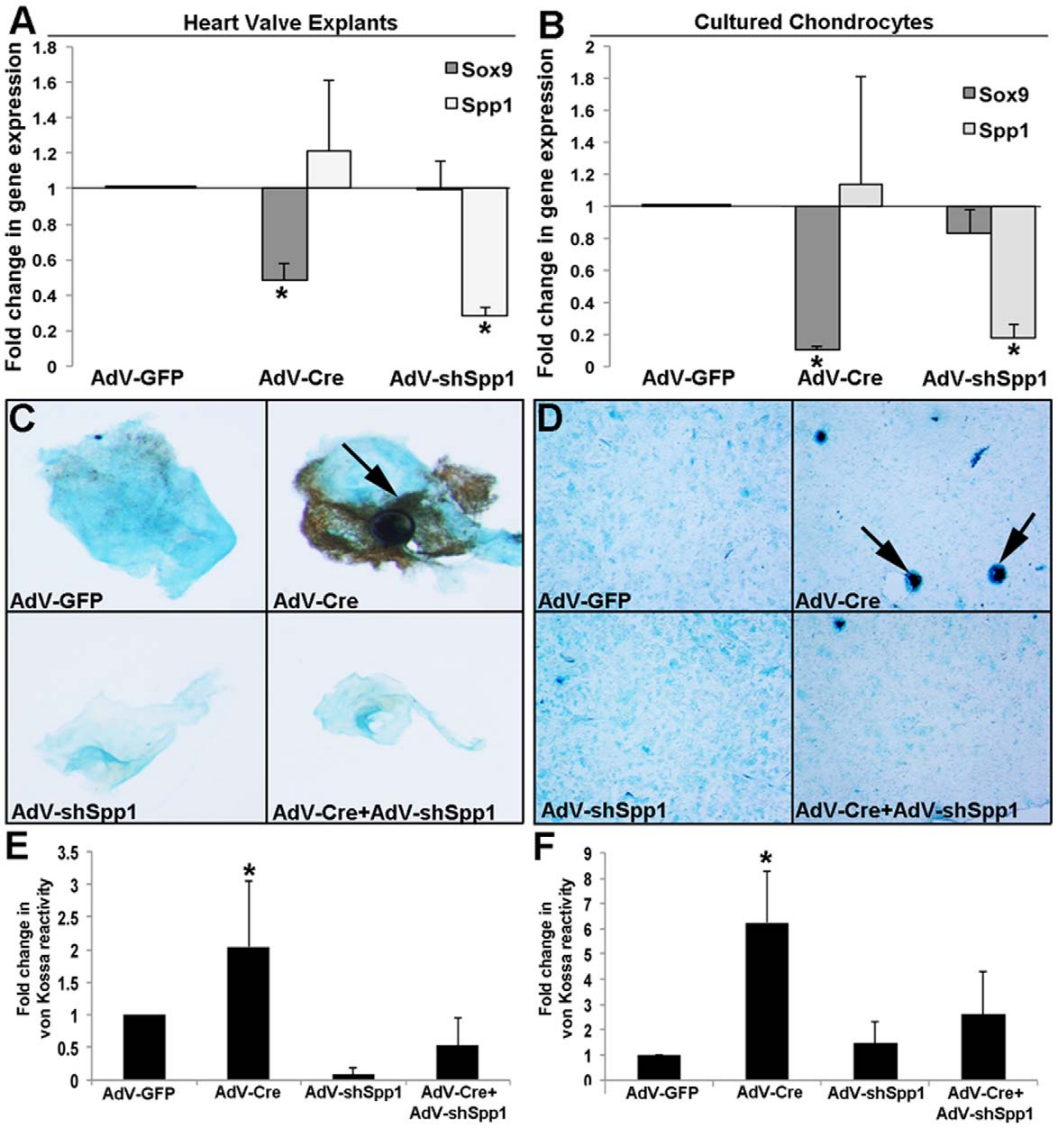
The requirement of Spp1 for Sox9-mediated matrix mineralization. Primary heart valve explants (A, C, E) and chondrocyte cultures (B, D, F) were infected with AdV-GFP, AdV-Cre or AdV-shSpp1 for 4 hours. In addition after 24 hours of infection, AdV-shSpp1 treated cultures were also treated with AdV-Cre. After 72 hours, RNA was isolated and qPCR performed to show expected *Sox9* and *Spp1* gene knockdown following AdV-Cre and AdV-shSpp1 infection respectively, compared to AdV-GFP. Alternatively, treated cultures were fixed and stained with von Kossa and alcian blue to detect mineralization and proteoglycan secretion respectively. (E, F) Quantitation of von Kossa reactivity normalized to area (alcian blue positive) in treated cultures compared to AdV-GFP controls. *Student's t−test<0.05 compared to AdV-GFP.

Previous studies in heart valves and limbs have shown that reduced *Sox9* function promotes premature matrix mineralization [21], [25]. To determine if *Spp1* is required for this *Sox9*-mediated process, heart valve explants and cultured rib chondrocytes isolated from post natal *Sox9^fl/fl^* mice were subject to infection with AdV-Cre, and/or an adenovirus containing a short hairpin (sh) RNA sequence to target *Spp1* knockdown (AdV-shSpp1). Compared to AdV-GFP controls, AdV-Cre and AdV-shSpp1 infection led to successful targeted knockdown of *Sox9* and *Spp1* respectively (Figure 5A, 5B). In addition, and consistent with our previous findings [25], AdV-Cre infection increased von Kossa reactivity in both heart valve explants (Figure 5C, 5E) and cultured chondrocytes (Figure 5D, 5F). However, the ability of AdV-Cre to promote von Kossa reactivity was significantly reduced in cultures pre-treated with AdV-shSpp1 (Figure 5C-5F). This suggests that Spp1 function is important for Sox9-mediated calcification in heart valves and chondrocytes.

## Discussion

The replacement of cartilage by mineralized tissue is imperative for development of long bones, ribs and vertebrae [1]. However, it is equally important that endochondral ossification does not occur in tissues that must remain cartilaginous, including hyaline cartilage and heart valves. Aberrations in this process can lead to ectopic matrix mineralization associated with heterotopic ossification of the skeletal system and calcification of heart valve tissue. Therefore it is important that a correct balance of cartilage and bone is established in the developing embryo and maintained in the adult. The transcription factor Sox9 has been shown to play pivotal roles in both these processes. While the positive role of Sox9 in cartilage formation has been well characterized [5], [6], [7], [8], [9], [10], its repressive function in osteogenic processes is less well understood. This study used a genome-wide approach along with supporting molecular assays and revealed a novel mechanism in which Sox9 interacts and negatively regulates the osteoblast-associated glycoprotein, *Spp1*. Our findings provide mechanistic insights into the pathological processes of matrix mineralization associated with human disease.

Calcific disease of the heart valves and vasculature has recently been described as an active, osteogenic-like process [26]. Despite our data suggesting that reduced *Sox9* function promotes matrix mineralization via relieved repression of *Spp1,* Osteopontin and Sox9 are both highly expressed in human valves excised from patients with end-stage calcific aortic valve disease [25], [33], [34], [35], [36], [37], [38]. However, as Osteopontin can be secreted by both osteoblasts and osteoclasts, it is not clear if high levels in calcified valves are associated with high affinity for hydroxyapatite to promote calcification [39], [40], or degradation of the mineralized matrix [41], [42], [43]. As our data suggests that *Spp1* function is required for matrix mineralization induced by Sox9 knockdown, it is suggested that high levels promote calcific processes in this system and reflect the osteogenic phenotype of pathological lesions [26]. However, while the sufficiency of *Spp1* to promote matrix mineralization in osteoblastic primary cells has previously been reported [28], further work is required to examine similar mechanisms in heart valves. In contrast to Osteopontin, it is not so clear why *Sox9* is overexpressed in late stages of calcific aortic valve disease. As our previous work has shown that reduced Sox9 function promotes calcification [25], it is speculated that increased Sox9 is a compensatory response to attenuate disease progression. Based upon findings in this study and others, this is likely at the level of repressing osteogenic signaling, and/or ‘re-activating’ developmental pathways to promote valve cell proliferation and differentiation processes, as previously observed in diseased valves [21], [44].

The observed repression of *Spp1* by Sox9 complements previous studies [16], [20] and provides an additional mechanism to explain the calcification phenotypes observed in *Sox9* mutant mice [13], [25]. However, further work is required to fully understand how Sox9 differentially regulates bone and cartilage target genes in the same system. One consideration is through the recruitment of transcriptional co-repressors and co-activators respectively. In cartilage, PGC-1α and CREB-binding protein associated with p300 form transcriptional complexes with Sox9 on the enhancer regions of chondrogenic genes to promote expression [45], [46]. However, it is not yet clear if these ‘activating’ complexes are absent on osteogenic target genes, or replaced by repressive machinery. In addition to considering loss of co-activators and recruitment of repressors, studies have also shown that Sox9 can evoke inhibitory responses on biological process through post-transcriptional modification. This includes negative regulation by PI3K/AKT [47], DNA methylation [48], as well as competition binding with other signaling proteins for SRY sites [49]. Based upon the reported complexity of Sox9 regulation, it is likely that defining molecular codes that determine Sox9 as an activator or repressor in connective tissue system will not be clear-cut. However, understanding more thoroughly how Sox9 mediates antagonism between cartilage and bone signaling pathways would have significant impact in understanding developmental and disease processes.

In summary, this current study highlights a novel mechanism by which Sox9 acts as a transcriptional repressor of *Spp1* to prevent matrix mineralization in heart valves and chondrocytes, in vitro. Despite these two connective tissue systems being functionally diverse, there is ever-increasing evidence to show that normal heart valve ECM shares molecular characteristics and signaling pathways with chondrogenesis [21], [23], [25]. Similarly, pathological calcification of heart valve ECM has recently been referred to as a bone-like process, although parallel ‘endochondral ossification’ events in valve interstitial cells have not been yet reported in human patients or mouse models of valve calcification. Nonetheless, our findings provide further support to show overlapping regulatory pathways between these two connective tissue systems and identify a previously unappreciated mechanism to prevent ossification in tissues that must remain cartilaginous for life-long function.

## Methods

### Chromatin Immunoprecipitation

#### ChIP-on-chip array

Sox9 chromatin immunoprecipitation (ChIP) was performed from post natal wild type mouse limb chromatin according to manufacturer's instructions (EZ-ChIP, Millipore) adapted for use with primary tissue lysates as follows. One neonate mouse forelimb per immunoprecipitation (IP) was diced and incubated in hypotonic lysis buffer for 30 minutes on ice and then vortexed for 10 s. Nuclear-enriched fraction was pelleted by centrifugation and resuspended in PBS containing 0.5% formaldehyde (Sigma) for 10 minutes. Chromatin shearing was achieved with 5, 10-second pulses, with a 10 second minimum refractory period using a benchtop sonicator (Misonix). IPs were performed using a total of 5μg of pooled polyclonal rabbit Sox9 antibodies (4 ug SC-17341 (Santa Cruz) and 1μg anti-Sox9 antibody (a kind gift from Dr. Michael Wegner), with 5μg of normal rabbit IgG used in parallel control experiments. Each ChIP was validated by polymerase chain reaction (PCR) to confirm enrichment of a known Sox9-bound promoter region in the *CLP* promoter [8]. Purified DNA from Sox9, and IgG control ChIP experiments was hybridized to a GeneChipMouse Promoter 1.0R Array (Affymetrix). Data analysis was performed with Tiling Analysis Software and Integrated Genome Browser (Affymetrix). Enrichment of target genes was determined by setting a graph threshold for signal intensity (value≥30) and minimum run (100 base pairs) in the Integrated Genome Browser (Affymetrix) based on a known Sox9 binding site in the *CLP* promoter.

#### ChIP-on-chip validation

Sox9 binding to the *Spp1* 5’ region observed in the ChIP-on-chip analysis was validated by quantitative real-time PCR in three independent experiments with post natal mouse limb lysates. Primers were designed to amplify 100–200 bp genomic DNA sequences surrounding the known SRY binding site within the promoter region of *CLP:* 5’-GAAAGAGGTAGCTTTTTAAT, 3’-AGCACCACCCTATTGTGGCT [8], in addition to SRY#1, SRY#2 and SRY#3 binding sites on *Spp1* as detected by the GeneChipMouse Promoter1.0R Array: SRY#1: 5’-CAATCTATCTCCATTGTCTGTC,3’-CTTTCTCTGAGATGCCTTCC;

SRY#2: 5’-AGCAGGGTTTGGCAAGTAGCACT 3’-TCCCGAAATGGAGAACACAGGCT; SRY#3: 5’-TGTATCCATGTGGCCTTTATC, 3’-GAGTAGATCACCCTCACAGAGAC. Real-time PCR was performed using a StepOnePlus Real Time PCR system (Applied Biosystems) with PefeCTA SYBR Green Fast Mix (Quanta) according to the manufacturer’s instructions. Real-time PCR data for ChIP enrichment is reported as fold increase over IgG, with significance calculated from fold changes using Student's t−test. p<0.05 was considered significant (n = 3).

### Luciferase Assays

The pGL3-*spp1p* construct was obtained from Dr. Paul Shore and contains a published 829 bp *Spp1* promoter sequence [30]. To generate pGL3-*spp1*En, a 1599 bp 5’ region of *Spp1* (Accession number AY220127) containing SRY#1 was amplified from genomic mouse tail DNA by PCR using the following primers 5’TAGGTACCCATTAAGCATTTAACT-TTTCCAGTGATG, and 3’TGAGCTCCTTCAGCAGAGGCAAGGAG, and ligated into pGL4 using introduced Sac1 and Kpn1 (Promega) sites. pGL3-*spp1* was produced by ligating the 1599 bp *spp1* enhancer region into the pGL3-*spp1p* plasmid using Kpn1 and Sac1 sites, 5’ of the *spp1* promoter sequence. Site-directed mutagenesis was used to target mutation of the SRY#1 consensus sequence using the following internal mutagenesis primers: 5’TGTCTATGGCATTTAAGCAGGGGATAAAA-GAATTT and 3’AAATTCTTTTA-TCCCCTGCTTAAATGCCATAGACA. This mutated fragment was similarly ligated 5’ of the *spp1* promoter sequence in pGL3-*spp1p*, to generate pGL3-*spp1*mut.

Luciferase assays were performed in HEK 293 and MC-3T3 cells (ATCC) plated at 2×10^5^ or 2×10^4^ per well of a 24-well plate respectively, 16–20 hours prior to transfection with Lipofectamine reagent (Invitrogen) according to manufacturer's instructions. 200 ng of pGL3-*spp1,* pGL3-*spp1*mut or pGL3 (200 ng/well), and pcDNA or pcDNA-Sox9 (200 ng/well), were co-transfected into each well, along with 20 ng of pGL4 (*Renilla* luciferase, Promega). All transfections were performed in 0.5 mL OptiMem for 4 hours before the addition of 0.5 mL DMEM (Sigma) supplemented with 4 mM L-Glutamine and 10% FBS. Cell lysates were collected 24 hours following transfection according to the manufacturer's instructions for dual luciferase assays (Promega). Data is represented as an average percent of luciferase activity of the pGL3-*spp1* co-transfected with pcDNA (set at 100%) and normalized to pGL4 *Renilla* signal (n = 3–8).

### Immunohistochemistry

Post natal mouse limbs and heart valves from E12.5, E18.5 and 3 month old adult mice were processed unfixed, through 10, 20 and 30% sucrose solutions before embedding in OCT freezing compound (Tissue-Tek) and sectioned at 10μm-thick. Frozen sections were post-fixed in 100% acetone for 10 minutes at −20°C and rinsed in 1xPBS prior to immunofluorescent staining as described [25] using antibodies against Sox9, a kind gift from Dr. Michael Wegner at 1∶1000 dilution, and Osteopontin (Santa Cruz sc-10593) at 1∶100 dilution. Following primary antibody incubation, sections were washed in 1xPBS and incubated with respective anti-rabbit and anti-goat Alexa 488 or 568 secondary antibodies at 1∶400/1xPBS dilution for 1 hour at room temperature, washed, and incubated with DAPI (blue) for identification of nuclei before mounting in Vectashield (Vector Laboratories).

### Primary Culture Systems

#### Heart valve explants

Mitral valve explants were isolated from post natal *Sox9^fl/fl^* mice and cultured as previously described [25]. Valve explant cultures were infected immediately after dissection with 1×10^10^ PFU/filter AdV-shSpp1 produced by Vector Biolabs using a commercially available *Spp1* targeting shRNA (174_0576-H-5, Open Biosystems) or control AdV-GFP [25]. Adenoviral infections were conducted in serum free medium for 4 hours before incubation in regular culture medium for 20 additional hours. 24 hours following the initial infections (AdV-shSpp1 or AdV-GFP), valve explants were treated with AdV-Cre (Vector Labs) or control Ad-GFP at 1×10^10^ PFU in serum free medium for 4 hours before incubation in regular culture medium for an additional 68 hours. This provided valve explants with loss of *Spp1* expression prior to *Sox9* knockdown, or infection with controls including *Spp1* knockdown only, *Sox9* knockdown only, or AdV-GFP. After 72 hours of infection, treated explants were removed for histological analysis [25] or RNA was extracted using standard Trizol protocols (Invitrogen) and cDNA was synthesized from 200 ng RNA using the qScriptcDNA Synthesis Kit (Quanta) for real-time PCR analysis.

#### Chondrocyte cultures

Primary chondrocytes were prepared from rib cartilage of postnatal day 1–3 *Sox9^fl/fl^* mice, according to an established protocol designed to maintain chondrocyte differentiation in monolayer culture [50]. Following 18 hours of culture, primary chondrocytes were infected with AdV-GFP or AdV-shSpp1 at 2.5×10^10^ PFU/10 cm^2^ in serum free medium for 4 hours before incubation in regular culture medium (DMEM supplemented with 2 mM L-Glutamine and 10% FBS) for an additional 20 hours. 24 hours following the initial infections (AdV-shSpp1 or AdV-GFP), chondrocytes were treated with AdV-Cre (Vector Labs) or control AdV-GFP at 2.5×10^10^ PFU/10 cm^2^ in serum free medium for 4 hours and then incubated in regular culture medium for an additional 68 hours. After 72 hours of infection, treated chondrocytes were fixed in 4% paraformaldehyde for histological analysis or RNA was extracted using standard Trizol protocols (Invitrogen) and cDNA was synthesized from 200 ng RNA using the qScriptcDNA Synthesis Kit (Quanta) for real-time PCR analysis. All animal procedures were approved by The University of Miami Institutional Animal Care and Use Committee (protocol # 09-171) and performed in accordance with institutional guidelines.

### Primary Cultures: Polymerase Chain Reaction

Quantitative real-time PCR was performed using a Step One Plus Real Time PCR system (Applied Biosystems) with the following TaqMan assays (Applied Biosystems): Human *18* *s* and mouse *Sox9, Spp1*, *CLP* and *Col2a1*. Reactions were performed using 10µl PerfeCTAFastMix (Quanta), 1µlTaqMan assay, 1µl cDNA, and 8µldeionized water per reaction. Real-time PCR for ChIP validation was performed using 10µl PerfeCTA SYBR Green FastMix (Quanta) with 0.5µl of each primer (at 20 pmol/µl), 1µl cDNA and 8µl deionized water per 20µl reaction. All reactions were performed with a 20 second activation step at 95°C followed by 40 cycles with 1 second denaturation at 95°C and a 20 second annealing/polymerization step at 60°C.

### Western Blotting

Protein lysates from treated chondrocyte cultures (1 well of a 6-well plate) were collected and lysed in 1× buffer (20 mM Tris (pH 7.5), 150 mM NaCl, 1 mM EDTA, 1 mM EGTA, 1% Triton X−100, 2.5 mM Sodium pyrophosphate, 1 mM β-glycerophosphate, 1 mM Na_3_VO_4_, supplemented with complete EDTA free protease inhibitor cocktail). 30μg of total protein was run on a 12% SDS PAGE gel and transferred to nitrocellulose membranes. Membranes were blocked in 5% fat free dried milk for 1 hr and probed with antibodies against Sox9 (1∶1000, 1 hr, Santa Cruz) and α-tubulin (1∶10,000, 1 hr, Chemicon/Millipore), followed by incubation with anti-rabbit-horseradish peroxidase (HRP)-conjugated secondary antibody (1∶10,000, 1 hr, Cell Signaling). Membranes were then washed three times in 1× TBST for 10 minutes. Western blots were developed using Super Signal West Femto Substrate (Pierce) and BioMax MR film (Eastman Kodak).

### Primary Cultures: Histology

Following culture and viral infection as described (Section 4.4), one mouse valve explant per filter was mounted and von Kossa staining was performed as described previously [25]. Chondrocyte cultures were stained following a similar protocol; cells were rinsed in PBS, fixed in 4% paraformaldehyde, washed 3 times in deionized water, and incubated in 5% silver nitrate solution for 1 hour under direct light (Schott Modulamp) in a reflective chamber. Chondrocytes were then washed in water, differentiated in 5% sodium thiosulfatepentahydrate for five minutes, and rinsed and counterstained for 20 minutes in 1% Alcian blue in 20% acetic acid. Quantification of von Kossa reactive area was performed using Image Pro Plus software and calculated as a percentage of von Kossa positive area (black) over total area indicated by alcian blue staining.

## Supporting Information

Table S1 **Data analysis of ChIP-on-chip using Tiling Analysis Software and Integrated Genome Browser (Affymetrix).** Enrichment of indicated target genes was determined by setting a graph threshold for signal intensity (value≥30) and minimum run (100 base pairs) in the Integrated Genome Browser (Affymetrix) based on a known Sox9 binding site in the *CLP* promoter.
(XLSX)
